# Current Evidence on Atypical Odontalgia: Diagnosis and Clinical Management

**DOI:** 10.1155/2012/518548

**Published:** 2012-07-09

**Authors:** Yoshihiro Abiko, Hirofumi Matsuoka, Itsuo Chiba, Akira Toyofuku

**Affiliations:** ^1^Division of Oral Medicine and Pathology, School of Dentistry, Health Sciences University of Hokkaido, 1757 Kanazawa, Ishikari-Tobetsu, Hokkaido 061-0293, Japan; ^2^Division of Disease Control & Molecular Epidemiology, School of Dentistry, Health Sciences University of Hokkaido, 1757 Kanazawa, Ishikari-Tobetsu, Hokkaido 061-0293, Japan; ^3^Department of Psychosomatic Dentistry, Faculty of Dentistry, Graduate School of Medical and Dental Science, Tokyo Medical and Dental University, Tokyo 113-8510, Japan

## Abstract

Patients with atypical odontalgia (AO) complain of medically unexplained toothache. No evidence-based diagnostic criteria or treatment guidelines are yet available. The present paper addresses seven clinical questions about AO based on current knowledge in the literature and discusses diagnostic criteria and guidelines for treatment and management. The questions are (i) What is the prevalence of AO in the community? (ii) What psychological problems are experienced by patients with AO? (iii) Are there any comorbidities of AO? (iv) Is local anesthesia effective for the relief of pain in AO? (v) Are there any characteristic symptoms of AO other than spontaneous pain? (vi) Are antidepressants effective for treatment of AO? (vii) Are anticonvulsants effective for treatment of AO? Our literature search provided answers for these questions; however, there is insufficient evidence-based data to establish guidelines for the diagnosis and treatment of AO. Overall, some diagnostic criteria for neuropathic pain and persistent dentoalveolar pain disorder may be applied to AO patients. The patient's psychogenic background should always be considered in the treatment and/or management of AO. The clinicians may need to treat AO patients using Patient-Oriented Evidence that Matters approach.

## 1. Introduction

Patients with atypical odontalgia (AO) complain of medically unexplained toothache. Symptoms include pain without any pathological changes and/or stronger pain than would be expected from the clinical findings. AO is a subgroup of persistent idiopathic facial pain disorder as defined by the International Headache Society [1]. Any dental procedures including scaling, restorative treatment, and endodontic treatment pose potential risk of AO. In many cases, the persistent nature of the pain prompts dentists to treat the teeth in the absence of any pathological findings. The treatment often exacerbates the pain instead of relieving it. Dentists usually consider a diagnosis of AO only after the failure of invasive treatment. Although it is crucial to establish diagnostic criteria for AO, the evidence-based literature to date has been insufficient to make this possible.

Several clinical management methods for AO have been reported [2–11]. The pathophysiological mechanism of AO may provide useful information for effective management. Possible pathophysiological mechanisms for AO include those with neuropathic, vascular, and psychogenic origins. Since the clinical course of AO varies among patients, and each case may stem from a different origin, a universal treatment method may not be effective for all AO patients, and it may be difficult to establish an evidence base for clinical management.

We formulated seven questions based on our clinical experience in the Japanese Society of Psychosomatic Dentistry. The questions are

- What is the prevalence of AO in the community?
- What psychological problems are experienced by patients with AO?
- Are there any comorbidities of AO?
- Is local anesthesia effective for the relief of pain in AO?
- Are there any characteristic symptoms of AO other than spontaneous pain?
- Are antidepressants effective for treatment of AO?
- Are anticonvulsants effective for treatment of AO?

These questions will be reviewed based on current knowledge in the literature, and diagnostic criteria and guidelines for treatment and management will be discussed.

## 2. What Is the Prevalence of AO in ****the Community? 

AO was found in 2.1% of a population of 3000 at the University of Southern California Orofacial Pain and Oral Medicine Center [12]. Other studies suggest that AO occurs in 3–6% of patients undergoing endodontic treatment [2, 13–17]. Polycarpou et al. [18] examined 175 patients at the Eastman Dental Hospital, a secondary healthcare facility, and found the prevalence of persistent pain after successful root canal treatments was 12%. They determined that significant risk factors leading to the development of chronic pain after endodontic treatment were the presence and duration of preoperative pain from the tooth, tenderness to percussion of the tooth preoperatively, female gender, previous painful treatment in the orofacial region, previous chronic pain problems, and whether surgical treatment was received. It is generally agreed that AO occurs more frequently in females than in males; 80–90% of all cases are female [7, 19–23]. Complaints were predominantly reported in the upper jaw (ratio 8 : 2) with the majority in the molar region (ratio 5 : 3) [20]. List et al. [24] reported in a study of 46 cases of AO that 56% of patients complained of pain in the upper jaw, compared with 45% in the lower jaw (one patient had pain in both jaws). These frequencies are useful for diagnosing AO after successful root canal treatment.

## 3. What Psychological Problems Are ****Experienced by Patients with AO?

Psychogenic pain is caused, increased, or prolonged by mental, emotional, or behavioral factors [25]. In addition to psychogenic pain, other pathophysiological mechanisms including neuropathic and vascular abnormalities have been linked with AO. Since the pain debut of 83% of AO patients was related to an invasive dental or surgical procedure [26], AO could be attributed to neuropathic pain caused by deafferentation [27–29]. However, pain in AO can also be spontaneous with no history of invasive dental or surgical procedures. Vascular pain has been proposed as a mechanism for AO, since AO patients occasionally suffer from migraine possibly caused by vascular events [30]. No scientific evidence has supported this hypothesis thus far [1, 31]. AO patients usually experience associated psychological problems such as depression, somatoform pain disorder, anxiety, demoralization, introversion, or hypochondriacal psychosis [6, 8, 21, 32–40]. AO patients may have a history of depression (66%) or depressive symptom (41%) [21, 41]. List et al. [26] examined 46 patients with AO and found significantly higher scores for depression and somatization in the AO group than the control group. Baad-Hansen et al. [42] found that mean depression and somatization scores were moderate to high in 46 patients with AO. These studies support a strong correlation between AO and psychological conditions. These psychological problems, however, may not reflect psychiatric diagnoses of AO. Takenoshita et al. [43] investigated 37 AO patients referred from psychiatric facilities to dental clinics and found that 33.3% of the patients had no specific psychiatric diagnosis. It is not known whether AO patients with no specific psychiatric conditions experience neuropathic and/or vascular pain. The psychiatric or psychological approach is generally effective but may not be absolutely essential for AO patients.

There is some argument as to whether psychological problems are the primary or secondary cause of AO [15, 42]. Graff-Radford [44] suggested that depression was not the cause of AO, and Brooke and Merskey [45] hypothesized that depression was not the sole cause of AO. These reports suggest that psychological factors are not significant in the genesis of AO [15]. Whether psychological problems are primary or secondary causes of AO, the psychological background of the patient should always be considered, given the high incidence of psychological problems in AO patients.

## 4. Are There Any Comorbidities of AO?

AO may share pain mechanisms with other chronic orofacial pain conditions, such as atypical facial pain and temporomandibular disorder (TMD). Baad-Hansen et al. [42] examined 46 AO patients and showed that episodic tension-type headache (TTH), chronic TTH, and myofascial temporomandibular disorder occurred in 46%, 18%, and 50% of AO patients, respectively. Other researchers reported that TMD pain, TTH, and widespread pain were significantly more common among AO patients than controls [26]. In addition to being linked with TMD or TTH, AO has been reported in patients with burning mouth syndrome (BMS) [11, 46–48]. However, no statistical data to date has established the frequency of the comorbidity of AO with BMS. TMD should be considered as a comorbid disorder for AO.

## 5. Is Local Anesthesia Effective for the Relief ****of Pain in AO?

Several investigators have studied the use of local anesthesia to relieve the pain of OA. Vickers et al. [10] reported a significant reduction in pain in a group of AO patients treated with local anesthesia. A randomized controlled trial undertaken by List et al. [5] corroborated the evidence of this paper. List et al. [5] evaluated the analgesic effect of local anesthesia (lidocaine) in a randomized controlled trial on 35 consecutive AO patients. They concluded that AO patients experienced significant, but not complete, pain relief from administration of local anesthetics compared with placebo. The pain relief was transient and was only at 15–120 min following the administration of the local anesthetics. In contrast, Graff-Radford and Solberg [49] found no significant difference in pain relief in AO patients after administration of local anaesthetic, although patients with nociceptive pain significantly improved. Two other uncontrolled studies showed similar findings [19, 21]. The clinical course of AO patients varies, and the effectiveness of local anesthesia may depend on the type of pathophysiological mechanisms of AO. Lidocaine patches were found to be clearly effective in reducing ongoing pain and allodynia in peripheral neuropathic pain syndrome [50]. Local anesthesia may transiently relieve pain in AO patients whose pain stems from neuropathic sources. Further investigations are needed to clarify this hypothesis.

## 6. Are There Any Characteristic Symptoms of AO Other Than Spontaneous Pain?

The persistent pain in AO can be altered by various stimuli. The sensations of 10 AO patients were compared with 10 matched healthy controls. The AO patients showed increased sensitivity to capsaicin and heat pain, but no significant differences in cold and mechanical sensitivity [13]. A study by Zagury et al. [51] comparing 21 AO patients with 18 controls found that the duration of pain sensation on cold application was significantly longer in AO patients, although the level of pain to cold stimulus was not statistically different between the AO and control group. List et al. [24] observed significant abnormalities in intraoral somatosensory function in AO patients, which may reflect peripheral and central sensitization of trigeminal pathways.

## 7. Are Antidepressants Effective for Treatment of AO?

**Figure 1:**
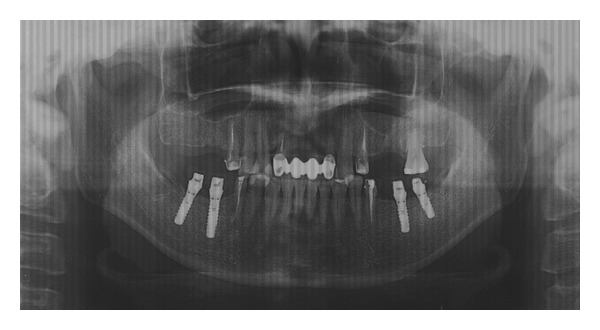
Panoramic radiograph shows a case of atypical odontalgia causing in a 57-year-old woman. The woman complained of toothache in the bilateral upper molars after the replacement of dental implant into the lower jaws. The root canal treatments of upper molars were performed, and the upper molars were finally extracted, since her pain was not relived by any treatments. A prescription of 40 mg per a day of amitriptyline decreased symptoms in the patient.

A meta-analysis of available data revealed that in patients diagnosed with psychogenic pain or somatoform pain disorder, antidepressant treatment resulted in a reduction in pain that was significantly greater than that of placebo [52]. Tricyclic antidepressants (TCAs) are the standard treatment choice for psychogenic pain or somatoform pain disorder. In the management of AO, TCAs have usually been prescribed alone or in combination with phenothiazines [2–4, 6–9, 11, 53, 54] (Figure 1). Adverse effects should be considered when prescribing both TCAs and phenothiazines. Phenothiazines have been linked with potentially irreversible adverse effects in the nervous system causing tardive dyskinesia. A literature review by Baad-Hansen et al. [42] noted that there is a need for a high-quality randomized controlled trial in AO patients to evaluate the effect of TCAs on AO pain. A recent study by Ito et al. [55] evaluated the effectiveness of milnacipran for pain relief in 36 patients with chronic pain in the orofacial region and a diagnosis of AO or burning mouth syndrome. They found that the visual analog scale score decreased significantly after treatment in comparison with the baseline score. To our knowledge, this is the only study to use milnacipran in the treatment of AO, although the effectiveness of milnacipran on other pain disorders has been established [56–62]. High-quality clinical research such as a randomized controlled trial should be performed to assess the effectiveness of milnacipran in the treatment of AO.

## 8. Are Anticonvulsants Effective for Treatment of AO?

Anticonvulsants have been recommended for the management of AO and orofacial pain by several authors [63–65]. Recently, a systematic review on the effectiveness of anticonvulsants for the management of orofacial pain was conducted using randomized controlled trials [66]. According to this paper, gabapentin [67], topical clonazepam [68], lamotrigine [69], and carbamazepine [70, 71] reduce the pain intensity significantly in patients with chronic masticatory myalgia, stomatodynia, refractory trigeminal neuralgia, and trigeminal neuralgia, respectively. There is, however, limited to moderate evidence supporting the efficacy of the anticonvulsants for treatment of patients with orofacial pain disorder. More randomized controlled trials were needed to confirm the effectiveness [66]. Although AO is a type of oral facial pain disorder, there is less evidence supporting their effectiveness for AO. According to a paper reviewed by Matwychuk [72], the anticonvulsants can be used as a treatment and diagnostic tool for trigeminal neuralgia, whereas antidepressants have been reported to show good results for atypical odontalgia. Anticonvulsants may be less effective for AO than antidepressants. Further investigation is needed to clarify this hypothesis.

## 9. Conclusion

There is insufficient evidence-based data available to establish guidelines for the diagnosis and treatment of AO thus far. Several diagnostic criteria for AO have been proposed [1, 6, 8, 9, 49]. Common findings in the criteria are “Pain is described as a toothache” and “No radiographic or laboratory investigations demonstrate relevant abnormality.” Many other findings are shown in the criteria, but may not be essential for diagnosis. The present clinical questions addressing prevalence, comorbid diseases, and the characteristics of the pain may be useful for the establishment of diagnostic criteria. Further investigations are needed to consolidate the evidence base.

Evidence-based data are also needed to establish guidelines for treatment and management of AO. Precise pathophysiological information about the underlying mechanisms involved in AO is needed to provide an effective strategy for treatment or management of AO. As mentioned above, the three main hypotheses about the pathophysiology of AO relate to whether the condition has a psychogenic, vascular or neuropathic derivation. Diagnostic criteria of defined neuropathic pain [73] and a treatment algorithm for neuropathic pain based on evidence-based research [74] are available. These criteria and the algorithm could be applied to AO patients whose pain can be attributed to neuropathic factors. Such patients may be identified by the fact that local anesthesia results in pain relief. Meier et al. [50] proposed that neuropathic mechanisms are the primary cause of AO pain, and that psychological disturbances are probably more a consequence than the cause of chronic pain. Recently, diagnostic criteria for persistent dentoalveolar pain disorder were proposed by International RDC-TMD Consortium [75]. These criteria may be adaptive for AO. Since there is a general consensus about the involvement of psychological problems as either a primary or secondary cause of AO, patients cannot be treated as if the pain is of neuropathic origin only. The patient's psychogenic background should always be considered in the treatment and/or management of AO. In the present paper, we attempted to use the most reliable evidence-based data. Recently, Patient-Oriented Evidence that Matters (POEM) is emerging as a more significant approach than evidence-based data for use in clinical practice [76, 77]. Given that in psychosomatic diseases, doctor-centered interventions, and close observation of the doctor-patient relationship are of particular importance [78], clinicians may need to treat AO patients using the POEM approach.
